# Association of biopsy core number and location with pain in patients undergoing a transperineal prostate biopsy under local anaesthesia: a secondary analysis of the APROPOS trial

**DOI:** 10.1097/JS9.0000000000000593

**Published:** 2023-08-01

**Authors:** Bi-Ming He, Qi-Wei Yang, Zhen-Kai Shi, Tang-Rao Ji, Shuai-Dong Wang, Hai Zhou, Zhi-Chao Jin, Zhi-Chao Yan, Jia-Jun Zhang, Hai-Feng Wang

**Affiliations:** a Department of Urology, Shanghai East Hospital, School of Medicine, Tongji University; b Department of Health Statistics, Naval Medical University, Shanghai; c Department of Urology, Lanxi People’s Hospital, Lanxi, People’s Republic of China

**Keywords:** pain, perineal nerve block, periprostatic block, transperineal prostate biopsy

## Abstract

**Background:**

APROPOS was a multicentre, randomized, blinded trial focus on investigating the perineal nerve block versus the periprostatic block in pain control for men undergoing a transperineal prostate biopsy. In the analysis reported here, the authors aimed to evaluate the association of biopsy core count and location with pain outcomes in patients undergoing a transperineal prostate biopsy under local anesthesia.

**Methods:**

APROPOS was performed at six medical centers in China. Patients with suspected prostate cancer were randomized to receive either a perineal nerve block or a periprostatic block (1:1), followed by a transperineal prostate biopsy. The secondary analysis outcomes were the worst pain experienced during the prostate biopsy and postbiopsy pain at 1,6, and 24 h.

**Results:**

Between 12 August 2020 and 20 July 2022, a total of 192 patients were randomized in the original trial, and 188 were involved in this analysis, with 94 patients per group. Participants had a median (IQR) age of 68 (63–72) and a median (IQR) prostate volume of 42.51 (30.04–62.84). The patient population had a median (IQR) number of biopsy cores of 15 (12–17.50), and 26.06% of patients had a biopsy cores count of more than 15. After adjusting the baseline characteristics, the number of biopsy cores was associated with the worst pain during the biopsy procedure in both the perineal nerve block group (*β* 0.19, 95% CI: 0.12–0.26, *P*<0.001) and the periprostatic block group (*β* 0.16, 95% CI: 0.07–0.24, *P*<0.001). A similar association was also evident for the postbiopsy pain at 1, 6, and 24 h. A lesser degree of pain in both groups at any time (r range −0.57 to −0.01 for both groups) was associated with biopsy cores from the peripheral zone of the middle gland, while other locations were associated with a higher degree of pain. In addition, the location of the biopsy core had less of an effect on pain during the biopsy (r range −0.01–0.25 for both groups) than it did on postbiopsy pain (r range −0.57–0.60 for both groups).

**Conclusions:**

In this secondary analysis of a randomized trial, biopsy core count and location were associated with pain in patients undergoing a transperineal prostate biopsy under local anesthesia. These results may be helpful for making clinical decisions about the anesthetic approach for scheduled transperineal prostate biopsies.

## Introduction

Highlights

- A higher biopsy cores count was associated with more pain.
- Biopsy cores on the peripheral zone of the middle gland were associated with less pain.
- Perineal nerve block was superior to prostatic block in pain control.

Prostate biopsy has been the gold standard for diagnosing prostate cancer in patients with an elevated serum prostate-specific antigen (PSA) or a suspicious digital rectal examination result and can be performed via either the transrectal route or the transperineal route^1^.

Although these two methods provide comparable cancer detection rates, the transperineal biopsy was called to replace the transrectal approach for its low infectious rate, even without antibiotic prophylaxis^2–4^. The European Association of Urology guidelines recommend performing a biopsy using the transperineal route whenever feasible^5^. However, the severe pain caused by a biopsy makes the procedure intolerable, preventing its widespread use^6^. Some local anesthesia methods for transperineal prostate biopsy were described, supporting pain relief and allowing the approach to be performed in the outpatient setting^7–9^. Although these methods have been preliminarily shown to be efficacious and safe, some limitations should be noted: first, comparative evidence for the different methods is limited; second, the findings have not been externally validated; and third, the factors influencing pain during transperineal biopsy have yet to be evaluated systematically.

To address these gaps in knowledge, we designed the APROPOS study, a multicentre and randomized controlled trial for comparing the perineal nerve block and the periprostatic block in patients undergoing transperineal prostate biopsy^10^. The primary analysis of the APROPOS study was presented previously, and clinically meaningful improvements in pain control were reported for the perineal nerve block compared with the periprostatic block^11^. In the current secondary analysis, we investigated the association of biopsy core number and location with pain outcome and obtained the following data.

## Methods

### Trial design and participants

This study is a secondary analysis of the APROPOS data set. The APROPOS trial was a multicentre, randomized, blinded, and parallel-group trial in which 192 patients suspected of having prostate cancer with or without a previous prostate biopsy between 12 August 2020 and 20 July 2022 were enrolled. Our analysis included 188 (97.9%) patients who had biopsy location details available. The key inclusion criteria included ages between 18 and 80 years old and an elevated PSA level between 4 and 20 ng/ml or/and suspicious rectal examination findings. The key exclusion criteria included a history of an allergy to the study drug, symptomatic acute/chronic prostatitis, and contraindications for a biopsy. The APROPOS trial was approved by the ethics committee with adherence to the Declaration of Helsinki. The trial was registered on ClinicalTrials.gov and all participants provided written informed consent. Additional information on the APROPOS trial has been published previously^11^. The trial was conducted and reported in accordance with the protocol and the statistical analysis plan (Supplementary Appendix, Supplemental Digital Content 1, http://links.lww.com/JS9/A810). The reporting of the study adheres to the CONSORT guidelines^12^ (Supplemental Digital Content 2, http://links.lww.com/JS9/A811) (Supplemental Digital Content 3, http://links.lww.com/JS9/A812).

In brief, included participants were assigned in a 1:1 proportion to undergo the perineal nerve block or periprostatic block, followed by a transperineal prostate biopsy. The random numbers were generated by the PROC PLAN statement of the SAS program by using block randomization. All participants underwent mpMRI before the prostate biopsy. The operator who performed the anesthesia was not involved in the subsequent biopsy procedure, pain assessment, data collection, or data analysis. The biopsy was performed by another urologist who was blinded to the allocation. All biopsy procedures used a freehand approach with 1–4 cores for targeted biopsy from each suspicious lesion shown on mpMRI and 8–24 cores for systematic biopsy. In addition, patients and the investigators involved in data analysis were blinded to the allocation. Other details about the APROPOS trial, such as the techniques of both types of blocks or sample size calculation, were shown in our previous study and the protocol (Supplementary Appendix, Supplemental Digital Content 1, http://links.lww.com/JS9/A810).

### Outcomes

The primary outcome of this study was the level of worst pain experienced during the biopsy procedure. The secondary outcomes were postbiopsy pain at 1, 6, and 24 h. Pain was measured by the NRS (numerical rating scale) from 0 to 10, where 0 represents no pain, and 10 represents the worst pain imaginable^13^.

### Statistical analysis

Categorical variables are described using frequencies and percentages, and between-group differences were tested by the *χ* ^2^-test or Fisher’s exact test. Continuous variables with a normal distribution are presented as the mean+/-SD and were compared using a *t*-test. Skewed variables are presented as medians (the first quartile, the third quartile) and were compared using the Wilcoxon rank-sum test.

A correlation analysis was conducted to evaluate the correlation between the number of biopsy cores and experienced pain at different time points for each biopsy location and each randomized group. Meanwhile, correlation coefficients were compared between the perineal nerve block and periprostatic block groups. Multiple linear regressions were conducted to explore the effect of the number of biopsy cores on pain experienced during biopsy and after biopsy, with adjustment for the randomized group, mean pain at baseline, age, BMI, PSA, prostate volume, PIRADS, and ASA. To explore the modification effect of the randomized group, an interaction between the randomized group and the number of biopsy cores was introduced in regression models, and *P*-values for interaction terms were reported. Stratification analyses by randomized groups were then conducted.

Statistical analyses and visualization were performed using SAS System for Windows (Version 9.4, SAS Institute Inc.) and R version 4.2.0 (R Foundation for Statistical Computing) with ggplot2 packages. Two-sided *P*-values of less than 0.05 were considered statistically significant.

## Results

### Patient population

We included 188 of the 192 APROPOS participants (97.9%) with available information and details of the biopsy location. Of those, the median (IQR) age was 68 (63–72), the median (IQR) prostate volume was 42.51 (30.04–62.84), the median (IQR) number of biopsy cores of the patient population was 15 (12–17.50), and 26.06% of patients had a biopsy core count of more than 15 (Table 1).

**Table 1:** Baseline characteristics.

Characteristic	Overall (*N*=188)	Perineal nerve block (*N*=94)	Periprostatic block (*N*=94)	*P*
Age, years	68.0 (63.0, 72.0)	69.0 (64.0, 74.0)	67.0 (62.0, 72.0)	0.043
BMI, kg/m^2^	24.05 (22.09, 25.35)	23.88 (21.77, 25.27)	24.18 (22.49, 25.53)	0.385
PSA	8.17 (5.82, 11.90)	8.67 (5.88, 12.73)	7.56 (5.59, 10.77)	0.191
Prostate volume	42.51 (30.04, 62.84)	44.43 (29.90, 63.00)	41.66 (31.39, 62.67)	0.938
Digital rectal examinations, positive	16 (8.51%)	6 (6.38%)	10 (10.64%)	0.296
PI_RADS				0.037
1–2	71 (37.77%)	44 (46.81%)	27 (28.72%)	
3	60 (31.91%)	24 (25.53%)	36 (38.30%)	
4	41 (21.81%)	19 (20.21%)	22 (23.40%)	
5	16 (8.51%)	7 (7.45%)	9 (9.57%)	
ASA				0.831
1	113 (60.11%)	58 (61.70%)	55 (58.51%)	
2	65 (34.57%)	32 (34.04%)	33 (35.11%)	
3	10 (5.32%)	4 (4.26%)	6 (6.38%)	
Number of biopsy cores	15.0 (12.0, 17.5)	15.0 (12.0, 16.0)	15.0 (12.0, 18.0)	0.230
<=15	139 (73.94%)	70 (74.47%)	69 (73.40%)	0.868
>15	49 (26.06%)	24 (25.53%)	25 (26.60%)	

Data are median (Q1,Q3) or *n* (%).

ASA, America Society of Anesthesiologists; PI-RADS, prostate imaging reporting and data system; PSA, prostate-specific antigen.

PI_RADS score range from 0 to 5, with higher scores indicating higher probability.

### Pain outcomes in different biopsy core count distributions

**Figure 1:**
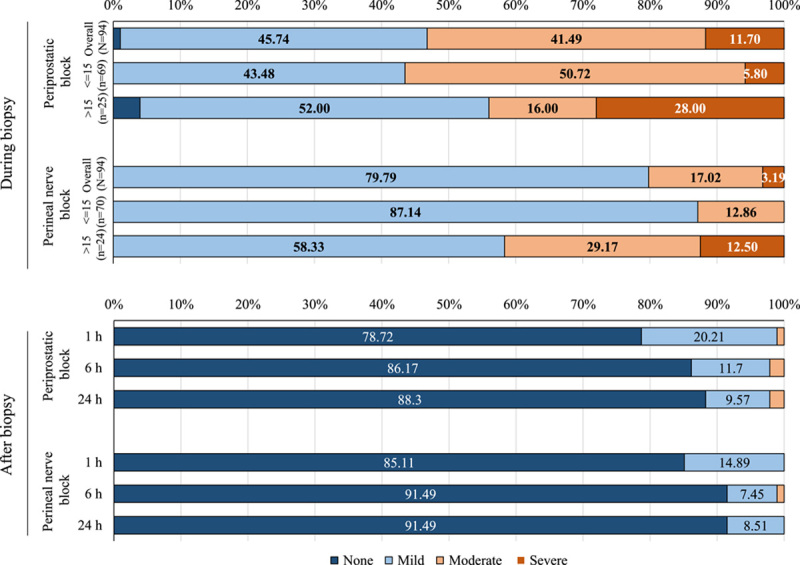
Relative distribution of pain on the stratified number of biopsy cores group. None pain is defined as a numerical rating scale (NRS) of 0; Mild pain is defined as an NRS of 1–3; Moderate pain is defined as an NRS of 4–6; Severe pain is defined as an NRS ≥7. In the ‘during biopsy’ section, the distribution of the worst pain scores was stratified by the number of biopsy scores (<=15, >15) and randomized group (perineal block and periprostatic block). Wilcoxon rank-sum test was applied to detect the difference in the distribution of the worst pain scores. Firstly, data were stratified by the randomized group for comparing <=15 and >15 subgroups. In the perineal block group, there was a statistically significant difference observed between the >15 biopsy scores subgroup (*n*=25) group and the <=15 biopsy scores subgroup (*n*=69), *P*=0.002. However, in the periprostatic group, no significant difference between the two groups was found (*P*=0.94). Secondly, data were stratified by the number of biopsy scores (<=15, >15) for comparing the perineal block subgroup and periprostatic subgroup. In the <=15 biopsy scores group, there was a statistical difference between the perineal block subgroup (*n*=70) and periprostatic subgroup (*n*=69), *P*<0.001. However, in the >15 biopsy scores group, the difference between the perineal block subgroup (*n*=25) and periprostatic subgroup (*n*=24) turned out to be nonsignificant, *P*=0.68.

Patients in the perineal nerve block group had a better outcome throughout the range of the NRS in unadjusted analysis than those in the periprostatic block group. When the number of biopsy cores was less than or equal to 15, the proportion of patients with mild pain (an NRS of 1–3) was 87.14% in the perineal nerve block group and 43.48% in the periprostatic block group. Similar results were observed when the number of biopsy cores was more than 15, with 12.50% severe pain (NRS≥7) in the perineal nerve block group compared with 28.00% in the periprostatic block group (Fig. 1). For different biopsy core count distributions of the same group, the pain was greater for those with more than 15 biopsy cores than for those with less than or equal to 15 biopsy cores during the biopsy. No significant differences were found between the two groups in postbiopsy pain at 1, 6, and 24 h.

### Biopsy core count-pain interaction

**Figure 2:**
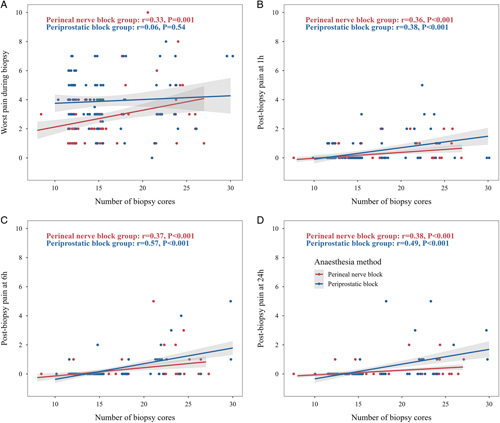
Pain plots for biopsy cores count estimated pain (solid line) with 95% CI (shade area) is shown during biopsy procedure (A), 1 h Postbiopsy (B), 6 h Postbiopsy (C) and 24 h Postbiopsy (D). The correlations between the number of biopsy scores and worst pain (during biopsy, postbiopsy at 1, 6, and 24 h) were estimated in the periprostatic and perineal block groups, respectively. The correlations in both groups were compared. No statistically significant differences were found between the groups for pain during biopsy (*P*=0.07; Fig. 2A), at 1 h postbiopsy (*P*=0.87; Fig. 2B), at 6 h postbiopsy (*P*=0.09; Fig. 2C), or at 24 h postbiopsy (*P*=0.36; Fig. 2D).

In unadjusted analyses, a higher biopsy core count was associated with worse pain during the biopsy procedure in the perineal nerve block group (r=0.33, *P*=0.001) but not in the periprostatic block group (r=0.06, *P*=0.54). A higher biopsy core count was associated with higher postbiopsy pain at 1, 6, and 24 h in both groups (in both groups, r ranged from 0.36 to 0.57, *P*<0.001) (Fig. 2).

After adjusting for baseline characteristics, the mixed-effects model indicated that for each additional biopsy core, the worst pain during the biopsy procedure rose by 0.19 (95% CI: 0.12–0.26, *P*<0.001) for the perineal nerve block and by 0.16 (95% CI: 0.07–0.24, *P*<0.001) for the periprostatic block (Table 2). This association was also evident in the level of postbiopsy pain at 1, 6, and 24 h, where the perineal nerve block group increased by 0.03 (95% CI: 0.01–0.06, *P*=0.013), 0.07 (95% CI: 0.03–0.10, *P*<0.001) and 0.03 (95% CI: 0.01–0.05, *P*=0.002), respectively, while the periprostatic block group increased by 0.07 (95% CI: 0.02–0.12, *P*=0.07), 0.14 (95% CI: 0.09–0.18, *P*=0.01), and 0.10 (95% CI: 0.06–0.15, *P*<0.001) (Table 2).

**Table 2:** Association between number of biopsy cores and pain experience during biopsy and after biopsy in different randomized group.

	Overall		Perineal nerve block group		Periprostatic block group		
Outcome	*β* (95% CI)^a^	*P* ^b^	*β* (95% CI)^a^	*P* ^b^	*β* (95% CI)^a^	*P* ^b^	*P* for interaction^c^
Worst pain during biopsy	0.18 (0.13–0.23)	<0.001	0.19 (0.12–0.26)	<0.001	0.16 (0.07–0.24)	<0.001	0.07
Postbiopsy pain at 1 h	0.05 (0.03–0.08)	<0.001	0.03 (0.01–0.06)	0.013	0.07 (0.02–0.12)	0.007	0.07
Postbiopsy pain at 6 h	0.10 (0.08–0.13)	<0.001	0.07 (0.03–0.10)	<0.001	0.14 (0.09–0.18)	<0.001	0.01
Postbiopsy pain at 24 h	0.07 (0.04–0.09)	<0.001	0.03 (0.01–0.05)	0.002	0.10 (0.06–0.15)	<0.001	<0.001

a Adjusted for Mean pain at baseline, age, BMI, PSA, Prostate volume, PIRADS, ASA.

b Estimated for *β*’s using regression.

c The interaction between the randomized group and the number of biopsy cores was introduced, significant interaction indicated the difference in *β*’s between the perineal nerve block group and periprostatic block group.

### Biopsy core location-pain interaction

**Figure 3:**
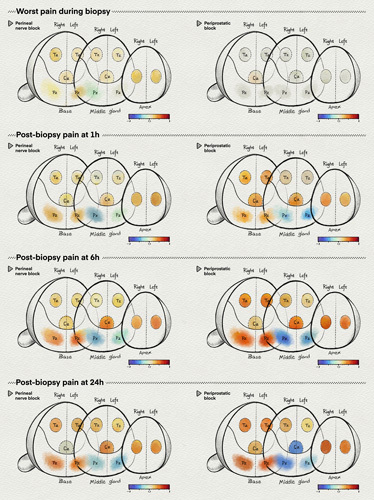
Prostate topographic maps of pain location correlates based on screen time. TZ, transition zone; CZ, center zone; PZ, peripheral zone.

The majority of biopsy cores were located in the middle gland of the prostate, accounting for 77.97% (12.07/15.48) in the perineal nerve block group and 76.17% (12.08/15.86) in the periprostatic block group (Table S1, Supplemental Digital Content 1, http://links.lww.com/JS9/A810). Correlation analysis between the number of biopsy cores and experienced pain at different time points for each biopsy location and each randomized group demonstrated that biopsy cores on the peripheral zone of the middle gland were associated with lower NRS in both groups at any time (r range −0.57 to −0.01 for both groups) (Table 3). Similar correlations were found in the further correlation analysis between biopsy core count and pain for location (no distinction between left and right) for the different groups (Table S2, Supplemental Digital Content 1, http://links.lww.com/JS9/A810); and between biopsy core count and pain for location (no distinction between left and right and the randomized group) (Table S3, Supplemental Digital Content 1, http://links.lww.com/JS9/A810). In addition, the location of the biopsy cores had less effect on pain during the biopsy (r range −0.01–0.25 for both groups) than it did on pain postbiopsy (r range −0.57–0.60 for both groups) (Table 3, Figs S1–S4 in the Supplementary Appendix, Supplemental Digital Content 1, http://links.lww.com/JS9/A810). To visualize these relationships, topographic maps of the r value between the pain and different locations of the prostate gland were produced and stratified into four groups of screen time (Fig. 3).

**Table 3:** Correlation between number of biopsy cores in different location and experienced pain at different time point.

		Worst pain during biopsy			Postbiopsy pain at 1 h			Postbiopsy pain at 6 h			Postbiopsy pain at 24 h		
Location	Average Number of biopsy	r1 (95% CI)	r2 (95% CI)	*P*	r1 (95% CI)	r2 (95% CI)	*P*	r1 (95% CI)	r2 (95% CI)	*P*	r1 (95% CI)	r2 (95% CI)	*P*
Apex													
Left	0.70	0.22 (0.02–0.41)	−0.02 (−0.23–0.18)	0.09	0.38 (0.19–0.54)	0.34 (0.15–0.51)	0.77	0.41 (0.23–0.57)	0.48 (0.31–0.62)	0.55	0.39 (0.20–0.55)	0.47 (0.30–0.62)	0.49
Right	0.70	0.23 (0.03–0.41)	0.05 (−0.15–0.25)	0.22	0.29 (0.09–0.46)	0.42 (0.23–0.57)	0.32	0.39 (0.21–0.55)	0.55 (0.40–0.68)	0.16	0.39 (0.21–0.55)	0.54 (0.38–0.67)	0.20
Middle gland													
Left peripheral zone	4.66	−0.01 (−0.21–0.20)	−0.06 (−0.26–0.15)	0.75	−0.16 (−0.35–0.05)	−0.47 (−0.62 to −0.30)	0.02	−0.29 (−0.47 to −0.09)	−0.41 (−0.57 to −0.23)	0.34	−0.37 (−0.54 to −0.18)	−0.34 (−0.51 to −0.15)	0.78
Right peripheral zone	4.54	−0.20 (−0.38–0.01)	−0.03 (−0.23–0.18)	0.24	−0.41 (−0.57 to −0.23)	−0.40 (−0.56 to −0.22)	0.93	−0.40 (−0.56 to −0.22)	−0.57 (−0.70 to −0.42)	0.12	−0.33 (−0.50 to −0.13)	−0.57 (−0.70 to −0.42)	0.03
Left transition zone	1.43	0.11 (−0.10–0.30)	−0.05 (−0.25–0.15)	0.27	0.15 (−0.05–0.34)	0.12 (−0.09–0.31)	0.82	0.20 (−0.00–0.39)	0.26 (0.06–0.44)	0.66	0.20 (−0.00–0.39)	0.20 (−0.00–0.39)	0.99
Right transition zone	1.44	0.15 (−0.05–0.35)	0.04 (−0.16–0.24)	0.44	0.07 (−0.14–0.27)	0.15 (−0.05–0.35)	0.55	0.18 (−0.02–0.37)	0.33 (0.13–0.50)	0.29	0.29 (0.09–0.46)	0.48 (0.31–0.62)	0.13
Central zone	0.56	0.19 (−0.01–0.38)	0.03 (−0.17–0.23)	0.27	0.35 (0.16–0.52)	0.40 (0.21–0.56)	0.72	0.40 (0.22–0.56)	0.51 (0.35–0.65)	0.35	0.40 (0.21–0.56)	0.52 (0.36–0.66)	0.29
Base													
Left peripheral zone	0.37	0.25 (0.04– 0.43)	0.05 (−0.15–0.25)	0.18	0.32 (0.13–0.49)	0.44 (0.26–0.59)	0.36	0.42 (0.24–0.58)	0.60 (0.46–0.72)	0.09	0.42 (0.24–0.57)	0.60 (0.46–0.72)	0.09
Right peripheral zone	0.38	0.18 (−0.02–0.37)	0.06 (−0.14–0.26)	0.39	0.36 (0.17–0.53)	0.43 (0.25–0.58)	0.59	0.40 (0.22–0.56)	0.58 (0.43–0.70)	0.11	0.40 (0.22–0.56)	0.58 (0.43–0.70)	0.10
Left transition zone	0.31	0.16 (−0.05–0.35)	0.02 (−0.18–0.22)	0.35	0.25 (0.05–0.43)	0.44 (0.26–0.59)	0.13	0.25 (0.05–0.43)	0.53 (0.37–0.66)	0.02	0.32 (0.12–0.49)	0.52 (0.36–0.65)	0.09
Right transition zone	0.38	0.24 (0.04–0.42)	−0.06 (−0.26–0.14)	0.04	0.27 (0.07–0.45)	0.39 (0.20–0.55)	0.39	0.28 (0.09–0.46)	0.50 (0.33–0.64)	0.09	0.36 (0.17–0.53)	0.51 (0.34–0.65)	0.22
Central zone	0.19	0.17 (−0.04–0.36)	0.15 (−0.05–0.34)	0.93	0.21 (0.01–0.40)	0.41 (0.22–0.56)	0.14	0.23 (0.03–0.41)	0.27 (0.07–0.45)	0.76	−0.00 (−0.21–0.20)	0.31 (0.11–0.48)	0.03

r1, correlation in perineal nerve block group; r2, correlation in periprostatic block group.

## Discussion

The main findings in this secondary analysis of the APROPOS trial were as follows: first, we found that a higher number of biopsy cores was significantly associated with increased pain scores, even after adjusted analysis. Second, biopsy cores on the peripheral zone of the middle gland were associated with less procedure pain, while other locations accounted for a higher pain score. Third, perineal nerve block was superior to periprostatic block in pain control for patients undergoing transperineal prostate biopsy.

To our knowledge, this was the first study designed to assess the association between the number and location of biopsy cores and pain in patients undergoing transperineal prostate biopsy with local anesthesia. The ideal local anesthesia would let the patient feel no pain during the biopsy procedure, regardless of the biopsy core count or location, as is the case with general or spinal anesthesia. Unfortunately, none of the currently reported forms of local anesthesia can be completely painless but can only reduce pain and make otherwise painful biopsy procedures tolerable^8,9,14,15^. Several factors, such as the biopsy core count, can naturally compromise the analgesic effect of this otherwise imperfectly adequate anesthesia. How the efficacy of local anesthesia varies with the number of biopsy cores has clinical implications for patient selection and the choice of treatment decision (e.g. local or general anesthesia). In typical clinical scenarios or clinical trials, the mode of a 10–12 core systemic biopsy combined with a 2–3 core targeted biopsy per suspicious lesion is primarily used^4,16,17^. Thereby, the number of biopsy cores for patients with only one suspicious lesion who undergo this mode of biopsy or patients who only undergo a systematic biopsy (patients with negative MRI results but high-level PSA or contraindications), and are most frequently encountered in the daily clinic is up to 15. For these patients, both perineal nerve block and periprostatic block were effective in pain control (none of the patients in the perineal nerve block group and only 5.8% in the periprostatic block group had severe pain during the biopsy procedure, and most of them felt no pain after the biopsy).

Pain increases with the increasing number of biopsy cores. When the number of biopsy cores was more than 15, 12.5% of patients in the perineal nerve block group and 28.0% in the periprostatic block group had severe pain. Although pain scores rose for patients in the perineal nerve block group, they were still in a good range overall, indicating that the perineal nerve block maintained good efficacy despite the increased number of biopsy cores. For the periprostatic block group, the raw data showed no significant increase in pain scores with the increase in the number of biopsy cores, but after adjustment, we further found a similar correlation between pain and the number of biopsy cores as observed in the perineal nerve block group. The postpain in both groups was much lower than the procedure pain. Importantly, this study’s median number of biopsy cores was 15, and the maximum number was 30, which indicates that the findings do not apply to some specific biopsy modes with high biopsy core counts (>30 cores), such as saturation biopsy or template-guided biopsy.

Further analysis based on biopsy location indicated that the location of biopsy cores on the peripheral zone of the middle gland is associated with less procedure pain, while other locations account for a higher pain score. This finding illustrates that with a fixed number and location of biopsy cores for a systematic biopsy, patients may suffer more pain when the targeted biopsy is performed in areas such as the apex, base, or transition zone of the prostate if the suspected lesion is in these areas. Notably, the two anesthetic methods had a similar location-pain relationship, but the perineal nerve group had a lower pain score than the periprostatic block group. We hypothesize a possible mechanism to explain this: the nerves blocked in the perineal nerve block group were closer to the trunk of the nerve innervating prostate pain than those blocked in the prostatic block group. It has two clinical implications: first, combining the two modalities may not substantially increase efficacy in pain control (even if it is easy and sounds feasible in practice) because both anesthetic modalities block nerves innervating similar areas of pain; second, there may be better anesthetic modalities that target nociceptive nerves innervating the anterior part of the prostate (or near the transition zone) and the base of the prostate than an anesthetic-enhancing combination of these modalities.

The correlation between biopsy core location and postpain was even more potent than its correlation with procedure pain. Such a stronger correlation at different time points after a biopsy helps us better identify and understand the relationship between pain and biopsy location. However, given that patients suffer much more pain, primarily intraoperative in origin and is substantially relieved after the procedure, this stronger correlation is less clinically important.

We acknowledge some limitations of our analysis. Although each of the outcomes assessed in this secondary analysis had been defined in the trial protocol, there was no prespecified statistical plan to assess the association between the number and location of biopsy cores and pain, which means that our findings should be considered hypothesis-generating.

In addition, as mentioned above, this study’s maximum number of biopsy cores was 30; thus, it is unclear how to translate the findings to those biopsy modes with a higher biopsy core count (>30). However, this limitation is outweighed by the less frequent use of biopsy with a high biopsy core in routine clinical practice. Although numerous transperineal prostate biopsy protocols have been reported, the protocol that combined a 10–12-core systematic biopsy with or without 2–3 cores targeted for each suspicious region of interest on MRI appears to be the most widely adopted. Some biopsy schemes are accompanied by a high number of cores, such as the template prostate mapping biopsy with a median of 49 cores taken. Such approaches have increased reported morbidities and lead to unpopularity in clinical scenarios, except for specific purposes that aim to accurately determine the whole gland disease status for selecting patients suitable for focal therapy^18,19^. Although such patients were not included in this study, based on the association between the number of cores and the pain found in this study, we speculate that patients who undergo this mode of biopsy will suffer more pain and may have difficulty tolerating the entire biopsy procedure. Hence, we recommend that such a biopsy should be taken under sedation or general anesthesia unless contrary evidence is found. Well-powered randomized trials are needed to characterize and ascertain our findings.

Last, this study did not involve some potential factors that may influence pain, such as anxiety. Reportedly, a higher level of anxiety was associated with severe biopsy pain^20^.

## Conclusions

The results of this secondary analysis of the APROPOS trial elucidated that a higher number of biopsy cores was associated with increased procedure pain in patients undergoing a transperineal prostate biopsy under local anesthesia. Additionally, our findings suggest that the location of biopsy cores was associated with procedural pain because biopsy cores on the peripheral zone of the middle gland were associated with less pain, while other locations accounted for more pain. Moreover, the perineal nerve block was superior to the periprostatic block in the efficacy of pain control in each biopsy core distribution. Notably, the findings of this study may not apply to patients scheduled to undergo biopsy modes with a biopsy core count of more than 30. The findings inform decisions about the anesthesia approach for a scheduled transperineal prostate biopsy, although further research will be helpful to confirm the conclusions.

## Ethical approval

The APROPOS trial was approved by the ethics committee (Shanghai East Hospital Ethics Committee, approval number: 2020050).

## Consent

Written informed consent was obtained from the patient for publication and any accompanying images. A copy of the written consent is available for review by the Editor-in-Chief of this journal on request.

## Sources of funding

This work was supported by Remedicine Co, grant 2019YFC0119100 from the National Key Research and Development Program of China, grant 81602220 from the National Natural Science Foundation of China, grant PWRd2020-17 from the Shanghai Pudong New District Health System Medical Talents Training Plan, China, grant PKX2020-S11 from the Fund of Development on Science and Technology of Shanghai Pudong New District, China, grant 18441910900 from the Shanghai Action Plan of Technological Innovation, grant 82002664 from the National Natural Science Foundation of China for Youth, grant No. 2020-QN-02 from Shanghai jiading district health commission scientific research project youth fund, and Meng Chao Talent Training Plan - Youth Research Talent Training Program of Eastern Hepatobiliary Surgery Hospital.

## Author contribution

R.-L.Z.: for depicted the figure; Q.H.: provided statistical assistance.

## Conflicts of interest disclosure

The authors declare no conflicts of interest.

## Research registration unique identifying number (UIN)

This trial is registered on ClinicalTrials.gov, NCT04501055.

## Guarantor

Haifeng Wang is the guarantor that accept full responsibility for the work and the conduct of the study, had access to the data, and controlled the decision to publish.

## Data availability statement

The data that support the findings of this study are available from the corresponding author, H.F. Wang, upon reasonable request. The data are not publicly available since this could compromise the privacy of research participants.

## Provenance and peer review

The paper was not invited.

## Supplementary Material

SUPPLEMENTARY MATERIAL
