# Supplementary material for: Association of biopsy core number and location with pain in patients undergoing a transperineal prostate biopsy under local anaesthesia: a secondary analysis of the APROPOS trial
Source: Int J Surg. 2023 Aug 1;109(10):3061–9. doi: 10.1097/JS9.0000000000000593 (PMC10583920; doi:10.1097/JS9.0000000000000593)
Supplement: SUPPLEMENTARY MATERIAL [file js9-109-3061-s001.pdf]

Supplementary appendix

List of supplementary material

Figure S1 Pain Plots for Biopsy Cores Count on different biopsy locations during prostate biopsy.

Figure S2 Pain Plots for Biopsy Cores Count on different biopsy locations post-prostate biopsy at 1 hour.

Figure S3 Pain Plots for Biopsy Cores Count on different biopsy locations post-prostate biopsy at 6 hours

Figure S4 Pain Plots for Biopsy Cores Count on different biopsy locations post-prostate biopsy at 24 hours

Table S1 The number of biopsy cores in different biopsy locations

Table S2 correlation between number of biopsy cores in different location (No distinction between left and right) and experienced pain at different time point.

Table S3 correlation between number of biopsy cores in different location (No distinction between left and right) and experienced pain at different time point. (No distinction between perineal nerve block and periprostatic block)

Table S4 Transperineal prostate biopsy protocol

Figure S1 – Pain Plots for Biopsy Cores Count on different biopsy locations during prostate biopsy.

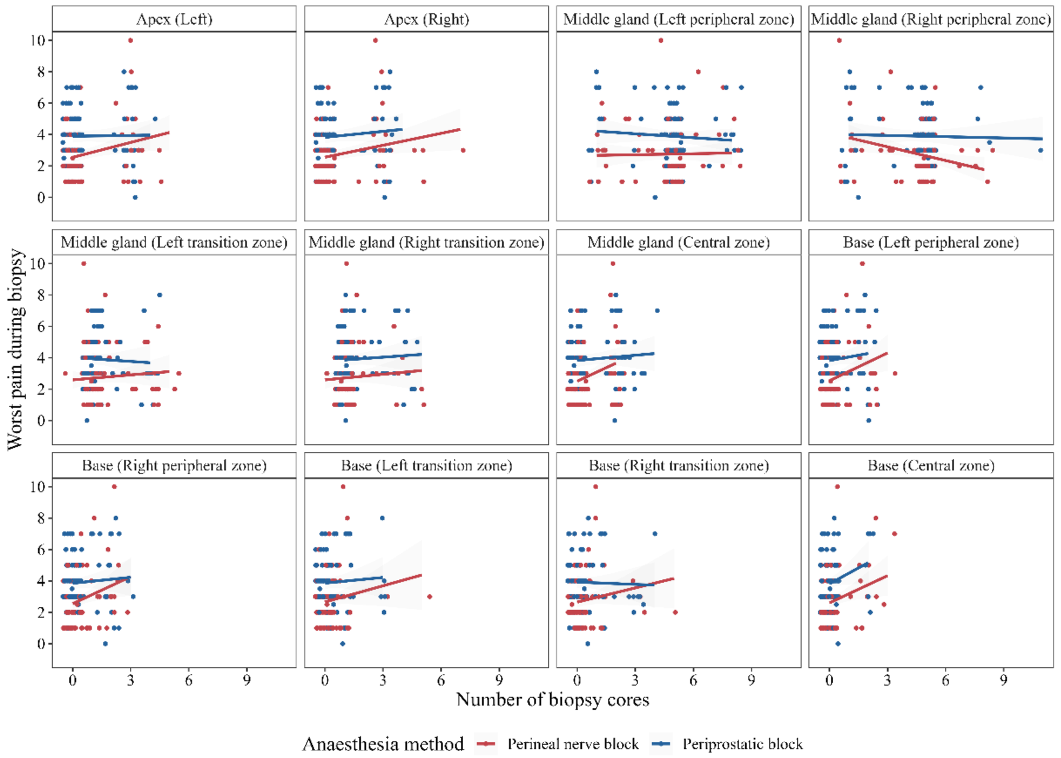

Figure S2 – Pain Plots for Biopsy Cores Count on different biopsy locations post- prostate biopsy at 1 hour.

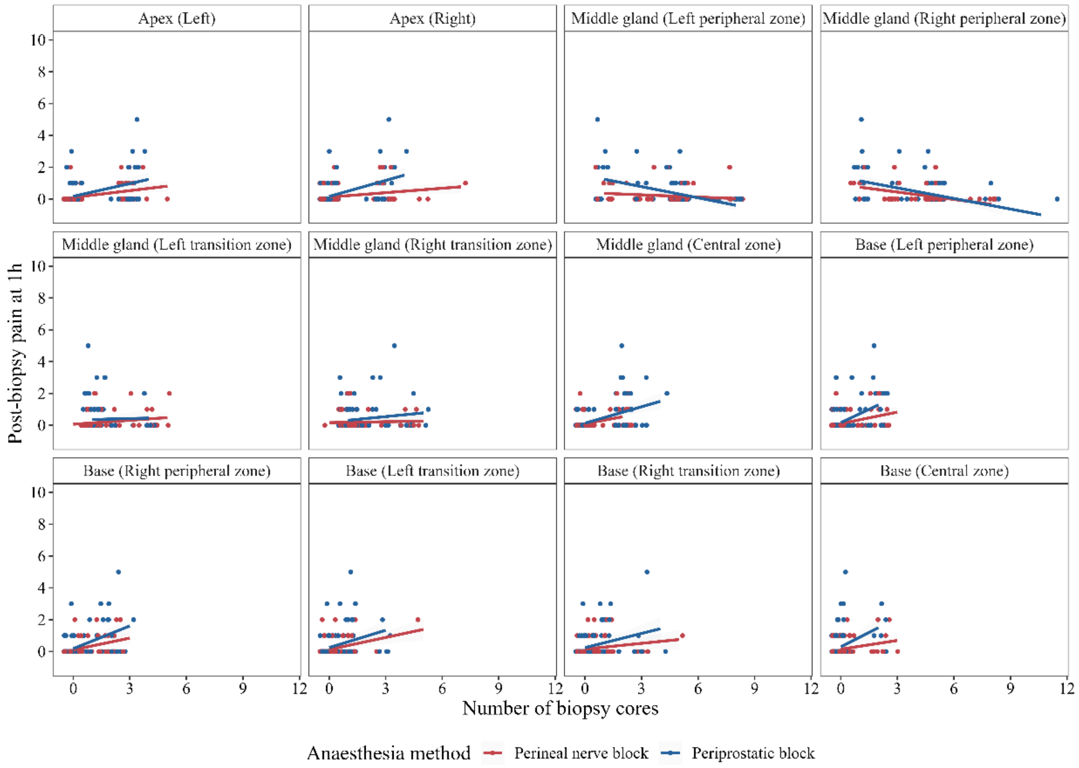

Figure S3 – Pain Plots for Biopsy Cores Count on different biopsy locations post-prostate biopsy at 6 hours

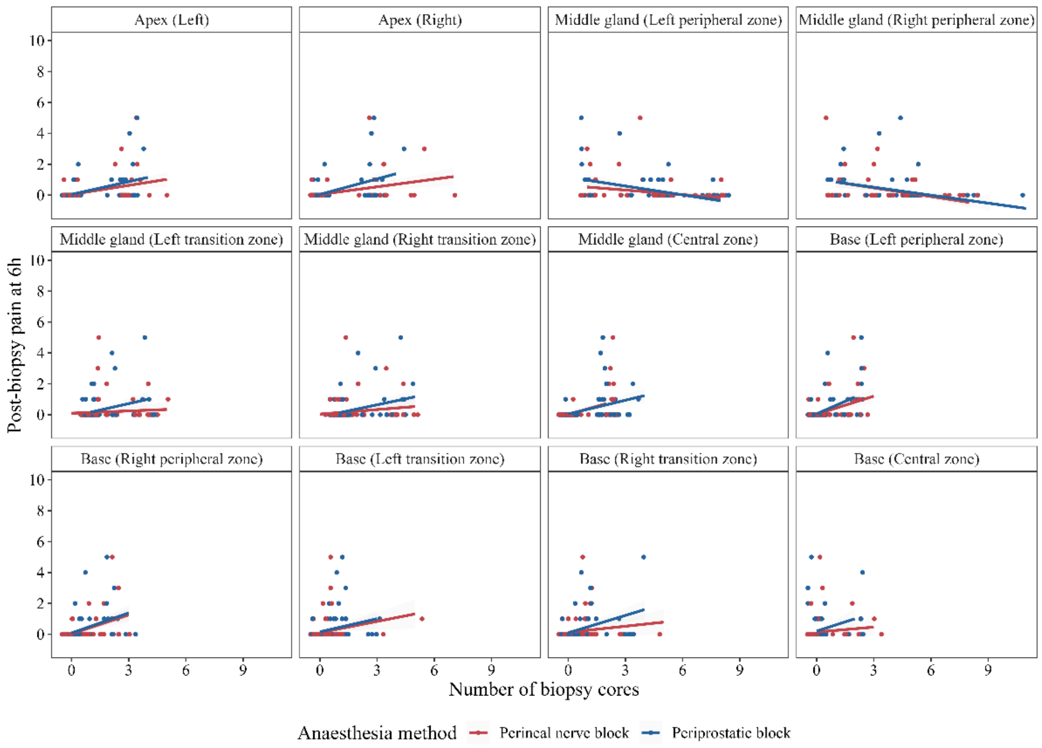

Figure S4 – Pain Plots for Biopsy Cores Count on different biopsy locations post-prostate biopsy at 24 hours

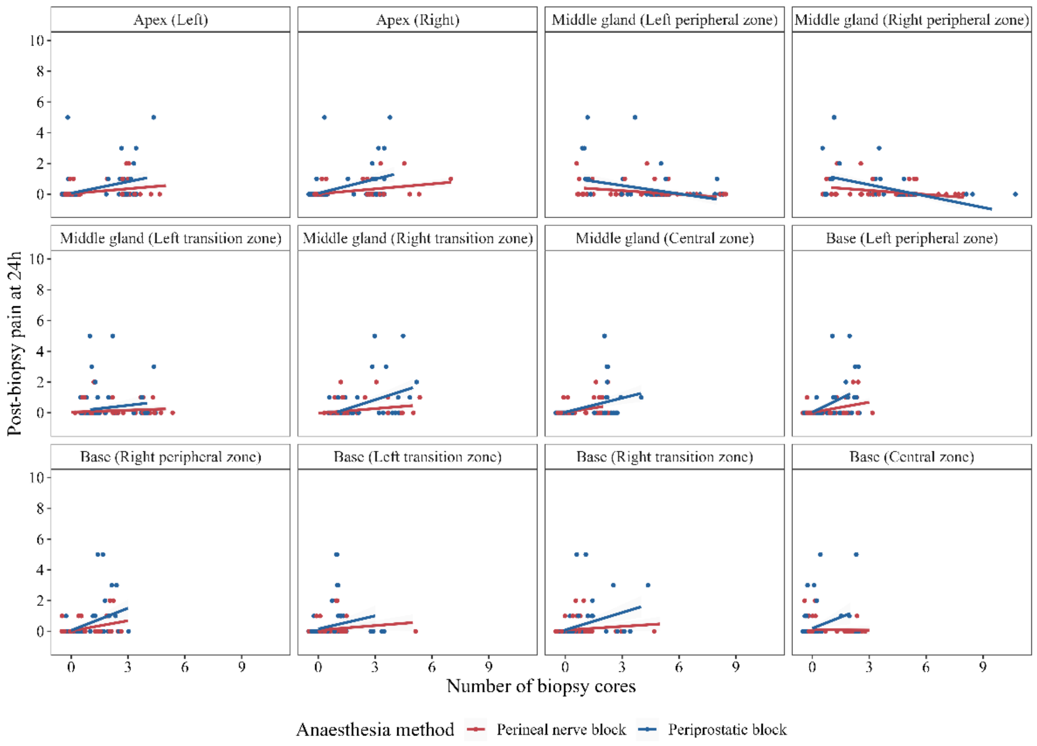

Table S1 The number of biopsy cores in different biopsy locations

	Perineal nerve block group		Periprostatic block group	
	Mean	Median	Mean	Median
Apex (Left)	0.64	0	0.76	0
Apex (Right)	0.77	0	0.63	0
Middle gland (Left peripheral zone)	4.59	5	4.74	5
Middle gland (Right peripheral zone)	4.61	5	4.48	5
Middle gland (Left transition zone)	1.52	1	1.34	1
Middle gland (Right transition zone)	1.35	1	1.52	1
Middle gland (Central zone)	0.43	0	0.7	0
Base (Left peripheral zone)	0.36	0	0.37	0
Base (Right peripheral zone)	0.35	0	0.4	0
Base (Left transition zone)	0.27	0	0.35	0
Base (Right transition zone)	0.33	0	0.44	0
Base (Central zone)	0.26	0	0.13	0

Table S2 correlation between number of biopsy cores in different location (No distinction between left and right) and experienced pain at different time point.

Location	Worst pain during biopsy			Post-biopsy pain at 1h			Post-biopsy pain at 6h			Post-biopsy pain at 24h		
	r1 (95% CI)	r2 (95% CI)	p	r1 (95% CI)	r2 (95% CI)	p	r1 (95% CI)	r2 (95% CI)	p	r1 (95% CI)	r2 (95% CI)	p
Apex	0.20(0.00, 0.39)	-0.00(-0.20, 0.20)	0.16	0.34(0.15, 0.51)	0.36(0.17, 0.52)	0.91	0.39(0.21, 0.55)	0.51(0.34, 0.64)	0.34	0.38(0.20, 0.54)	0.50(0.33, 0.63)	0.35
Middle gland												
Peripheral zone	-0.08(-0.28, 0.13)	0.01(-0.20, 0.21)	0.57	-0.28(-0.45, -0.08)	-0.42(-0.57, -0.24)	0.27	-0.30(-0.47, -0.10)	-0.45(-0.60, -0.28)	0.22	-0.36(-0.53, -0.17)	-0.43(-0.58, -0.25)	0.59
Transition zone	0.12(-0.09, 0.31)	-0.05(-0.25, 0.15)	0.26	0.15(-0.05, 0.35)	0.11(-0.09, 0.31)	0.77	0.21(0.01, 0.40)	0.37(0.19, 0.54)	0.24	0.24(0.04, 0.42)	0.46(0.28, 0.60)	0.09
Central zone	0.19(-0.01, 0.38)	0.03(-0.17, 0.23)	0.27	0.35(0.16, 0.52)	0.40(0.21, 0.56)	0.72	0.40(0.22, 0.56)	0.51(0.35, 0.65)	0.35	0.40(0.21, 0.56)	0.52(0.36, 0.66)	0.29
Base												
Peripheral zone	0.20(0.00, 0.39)	0.05(-0.15, 0.25)	0.29	0.36(0.17, 0.52)	0.43(0.25, 0.58)	0.57	0.41(0.22, 0.56)	0.58(0.43, 0.70)	0.12	0.40(0.22, 0.56)	0.58(0.43, 0.70)	0.11
Transition zone	0.21(0.01, 0.40)	-0.07(-0.27, 0.13)	0.05	0.25(0.05, 0.43)	0.36(0.17, 0.53)	0.4	0.27(0.07, 0.44)	0.47(0.30, 0.62)	0.1	0.32(0.13, 0.49)	0.47(0.29, 0.61)	0.24
Central zone	0.17(-0.04, 0.36)	0.15(-0.05, 0.34)	0.93	0.21(0.01, 0.40)	0.41(0.22, 0.56)	0.14	0.23(0.03, 0.41)	0.27(0.07, 0.45)	0.76	-0.00(-0.21, 0.20)	0.31(0.11, 0.48)	0.03

r1, correlation in perineal nerve block group; r2, correlation in periprostatic block group.

Table S3 correlation between number of biopsy cores in different location (No distinction between left and right) and experienced pain at different time point. (No distinction between perineal nerve block and periprostatic block)

Location	Worst pain during biopsy		Post-biopsy pain at 1h		Post-biopsy pain at 6h		Post-biopsy pain at 24h	
	r (95% CI)	p	r (95% CI)	p	r (95% CI)	p	r (95% CI)	p
Apex	0.09(-0.05, 0.23)	0.21	0.35(0.21, 0.47)	<0.001	0.45(0.33, 0.56)	<0.001	0.44(0.32, 0.55)	<0.001
Middle gland								
Peripheral zone	-0.02(-0.16, 0.13)	0.83	-0.35(-0.47, -0.22)	<0.001	-0.38(-0.50, -0.25)	<0.001	-0.40(-0.51, -0.27)	<0.001
Transition zone	0.03(-0.11, 0.17)	0.66	0.13(-0.01, 0.27)	0.07	0.30(0.16, 0.42)	<0.001	0.35(0.22, 0.47)	<0.001
Central zone	0.14(0.00, 0.28)	0.05	0.39(0.26, 0.50)	<0.001	0.48(0.36, 0.58)	<0.001	0.48(0.36, 0.58)	<0.001
Base								
Peripheral zone	0.11(-0.03, 0.25)	0.13	0.39(0.26, 0.51)	<0.001	0.50(0.39, 0.60)	<0.001	0.50(0.39, 0.60)	<0.001
Transition zone	0.08(-0.07, 0.22)	0.3	0.32(0.19, 0.44)	<0.001	0.39(0.26, 0.50)	<0.001	0.41(0.28, 0.52)	<0.001
Central zone	0.11(-0.03, 0.25)	0.12	0.28(0.14, 0.41)	<0.001	0.23(0.09, 0.36)	0.002	0.13(-0.01, 0.27)	0.08

r, correlation (included both perineal nerve block group and periprostatic block group)

Table S4 Transperineal prostate biopsy protocol

Centre	Systematic biopsy		Targeted biopsy				Systematic biopsy combined with targeted biopsy
	The number of cores	Men underwent systematic biopsy	The number of cores for each suspicious lesion	Total number of cores for targeted biopsy	method	Men underwent targeted biopsy	Total number of cores
**Centre1**	12	90 of 90	3	3~6	cognitive fusion	55 of 90	12~18
**Centre2**	8~24 (based on the prostate volume)	40 of 40	3~5	3~13	cognitive fusion or software fusion (Koelis Trinity fusion biopsy system)	28 of 40	13~30
**Centre3**	8~12 (based on the prostate volume)	42 of 42	1~3	1~3	cognitive fusion	23 of 42	8~15
**Centre4**	12	8 of 8	3	3~6	cognitive fusion	5 of 8	12~18
**Centre5**	12	6 of 6	3	3	cognitive fusion	4 of 6	12~15
**Centre6**	12	6 of 6	3~4	3~4	cognitive fusion	3 of 6	12~16
