# Supplementary figures and images for: Association of biopsy core number and location with pain in patients undergoing a transperineal prostate biopsy under local anaesthesia: a secondary analysis of the APROPOS trial
Source: Int J Surg. 2023 Aug 1;109(10):3061–9. doi: 10.1097/JS9.0000000000000593 (PMC10583920; doi:10.1097/JS9.0000000000000593)

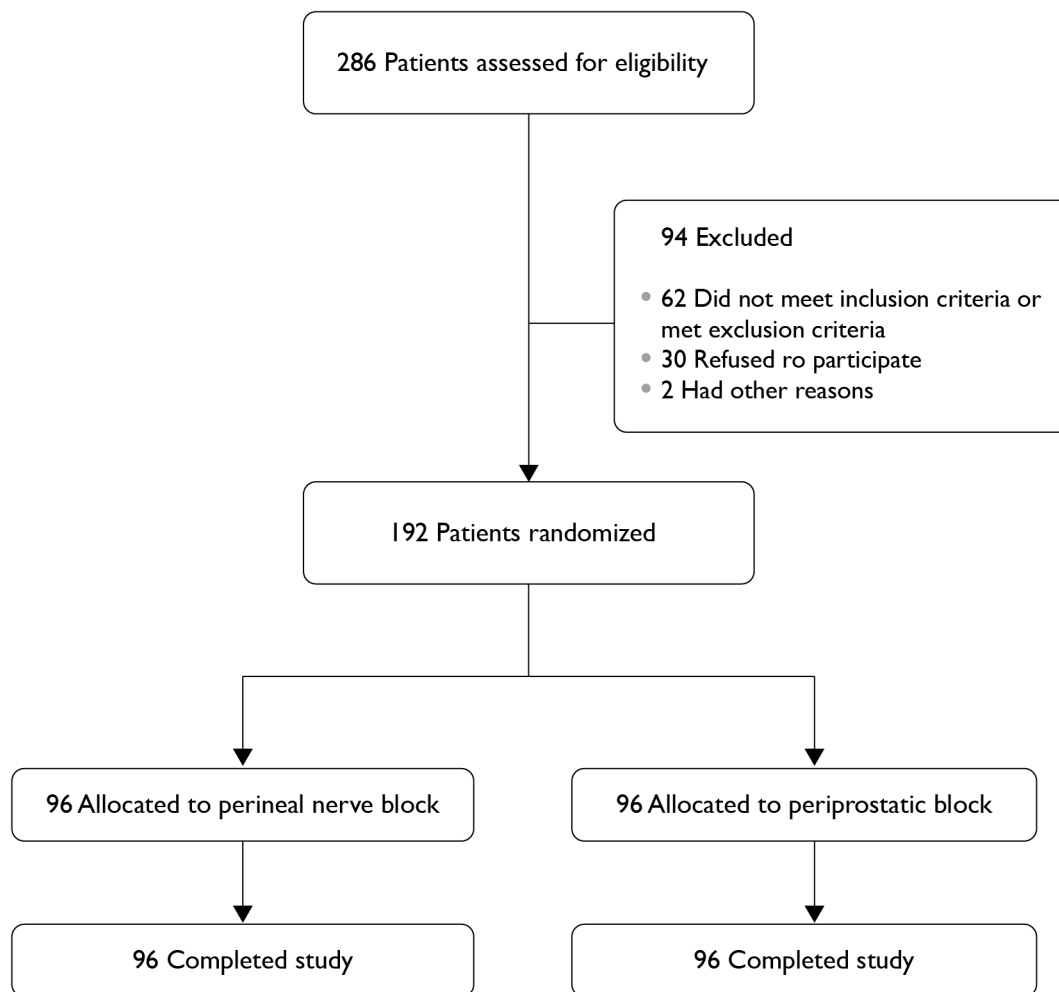

Supplement: SUPPLEMENTARY MATERIAL [file js9-109-3061-s003.pdf]
